# The use of a robotic tibial rotation device and an electromagnetic tracking system to accurately reproduce the clinical dial test

**DOI:** 10.1007/s00167-016-4042-0

**Published:** 2016-02-18

**Authors:** S. K. Stinton, R. Siebold, H. Freedberg, C. Jacobs, T. P. Branch

**Affiliations:** University Orthopedics, 441 Armour PL NE, Decatur, GA 30324 USA; Institute for Anatomy and Cell Biology, Ruprecht-Karls-University, Heidelberg, Germany; HKF – International Center for Hip, Knee, Foot Surgery and Sportstraumatology, ATOS Klinik, Heidelberg, Germany; Suburban Orthopaedics, Bartlett, IL USA; University of Kentucky, Lexington, KY USA; Morehouse College, Atlanta, GA USA

**Keywords:** Rotatory instability, Dial test, Knee laxity, Robotic knee testing, Posterolateral corner

## Abstract

**Purpose:**

The purpose of this study was to: (1) determine whether a robotic tibial rotation device and an electromagnetic tracking system could accurately reproduce the clinical dial test at 30° of knee flexion; (2) compare rotation data captured at the footplates of the robotic device to tibial rotation data measured using an electromagnetic sensor on the proximal tibia.

**Methods:**

Thirty-two unilateral ACL-reconstructed patients were examined using a robotic tibial rotation device that mimicked the dial test. The data reported in this study is only from the healthy legs of these patients. Torque was applied through footplates and was measured using servomotors. Lower leg motion was measured at the foot using the motors. Tibial motion was also measured through an electromagnetic tracking system and a sensor on the proximal tibia. Load-deformation curves representing rotational motion of the foot and tibia were compared using Pearson’s correlation coefficients. Off-axis motions including medial–lateral translation and anterior–posterior translation were also measured using the electromagnetic system.

**Results:**

The robotic device and electromagnetic system were able to provide axial rotation data and translational data for the tibia during the dial test. Motion measured at the foot was not correlated to motion of the tibial tubercle in internal rotation or in external rotation. The position of the tibial tubercle was 26.9° ± 11.6° more internally rotated than the foot at torque 0 Nm. Medial–lateral translation and anterior–posterior translation were combined to show the path of the tubercle in the coronal plane during tibial rotation.

**Conclusions:**

The information captured during a manual dial test includes both rotation of the tibia and proximal tibia translation. All of this information can be captured using a robotic tibial axial rotation device with an electromagnetic tracking system. The pathway of the tibial tubercle during tibial axial rotation can provide additional information about knee instability without relying on side-to-side comparison between knees. The translation of the proximal tibia is important information that must be considered in addition to axial rotation of the tibia when performing a dial test whether done manually or with a robotic device. Instrumented foot position cannot provide the same information.

**Level of evidence:**

IV.

## Introduction

The dial test was developed for the evaluation of a posterolateral corner (PLC) injury in the knee [5, 6, 15, 16]. The traditional description of the test includes rotation of the foot, while in dorsiflexion, from neutral to full external rotation and from neutral to full internal rotation while noting the angle created by the foot and the thigh. An increased amount of external rotation on the injured side is indicative of a potential PLC injury [6]. This simplistic view of the dial test does not reflect the sophisticated capture of information during the test [1]. The modern dial test involves the patient in the supine position or seated with the knee at 30° or 90° of flexion. The clinician rotates the tibia along its long axis both internally and externally while noting the position of the tibial tubercle and the force necessary to rotate the tibia. An instrumented dial test must reproduce these exact characteristics and must be able to quantify them.

An injury to the PLC is a complex injury that involves damage to multiple structures including but not limited to: the arcuate ligament, the popliteus tendon, the lateral meniscus, the lateral collateral ligament and the posterior cruciate ligament [19, 21, 22]. Only extreme damage creates biomechanical changes obvious to the clinician during a manual clinical knee examination [5]. In fact, isolated increased external tibial axial rotation may not identify the patient with a PLC injury without additional manoeuvres during the examination [13, 14]. A description of rotatory instability by measuring the relative translation of the lateral compartment of the knee with respect to the medial compartment appears to provide better accuracy in the diagnosis of a PLC injury [7, 25].

Instrumented devices have been developed in an attempt to better record the complex tibial motion during the dial test [4, 24, 27, 28]. However, these devices still have measurement error due to variability in the applied force, strain rate and patient setup. A robotic testing device has been previously developed that allows for consistent patient setup and consistent application of force [9, 11]. This device can generate load-deformation curves to describe tibial motion using the torque and rotation data collected through the motor. An improved version of this robotic testing device in which rotational data was collected in six degrees of freedom using an electromagnetic tracking system has been developed [8, 10]. To date, only long-axis rotation data has been reported using that system. However, long-axis rotation alone is not adequate to describe the true motion of the tibia during the dial test.

The purpose of this study was: (1) to determine whether a robotic tibial rotation device and an electromagnetic tracking system could accurately reproduce the clinical dial test at 30° of knee flexion; (2) to compare rotation data captured at the footplates of the robotic device to tibial rotation data measured using an electromagnetic sensor on the proximal tibia. The primary hypothesis of the study was that the robotic device and electromagnetic system would be able to provide data that would accurately represent the motion of the tibia during the dial test. A second hypothesis was that motion data measured using servomotors attached to footplates of the robotic device would not accurately represent the motion of the tibia as measured using an electromagnetic sensor on the proximal tibia. A final hypothesis was that the relationship between medial–lateral and anterior–posterior translation during tibial axial rotation could provide additional valuable information to the clinician.

## Materials and methods

After obtaining ethics approval, a subset of 68 patients from a cohort study following single-bundle (SB) and double-bundle (DB) anterior cruciate ligament (ACL) reconstruction using hamstring grafts were included in the study. Upon entrance into the study, patients signed human investigations consent for this IRB-approved study per the independent review board (Landesärztekammer Baden-Württemberg Ethics Commission: ID# 051-06-f). For the purpose of this paper, only the healthy knees of the patients were analysed. At follow-up, two patients were identified as having an injured opposite extremity through clinical examination, instrumented laxity testing or magnetic resonance imaging and were excluded, and another patient did not attend the follow-up. All of the remaining 65 patients had a unilateral knee injury with the opposite knee described as healthy with no history of injury or symptoms. Of these 65 patients, 33 had both knees instrumented with an electromagnetic motion tracking system (Flock of Birds, Ascension Technologies, a subdivision of NDI, Bakersfield, CA, USA) for analysis of proximal tibial motion. One patient had bilateral ACL reconstructions and was eliminated from the study group. The data from the healthy knees of the 32 remaining patients were included in this study.

**Fig. 1:**
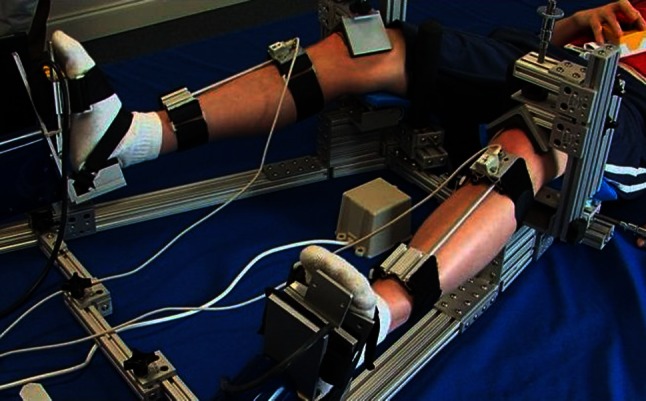
Robotic testing device setup with the electromagnetic tracking system

Each patient was evaluated using a robotic tibial axial rotation testing device and an electromagnetic tracking system. The robotic testing device consisted of two servomotors designed to apply torque about the centre of rotation of the tibia (Fig. 1). The system and patient setup were identical to that used in previous studies [6, 8]. Patients were positioned supine in the device, with both knees flexed to 30° and with both feet attached to footplates that were mounted to the servomotor system. To limit ankle motion during rotational testing, the foot was maximally dorsiflexed using inflatable air bags at the level of the metatarsal heads.

The second toe was positioned perpendicular to the floor. The position of the toe was verified using a digital goniometer referencing the earth. This was considered 0 rotation for this study with internal rotation and external rotation defined as such.

An electromagnetic sensor was used to track the motion of the tibia during testing. The electromagnetic tracking system could record the position of the tibia with respect to the femur with an accuracy of 0.48 mm and 0.30° based on root mean square error (0.88 mm and 0.48° 95 % CI). The sensor was attached to a custom aluminium harness that did not interfere with the electromagnetic tracking system. The sensor was calibrated such that the *x*-axis was directed along the long axis of the harness, the *y*-axis was directed laterally and the *z*-axis was directed posteriorly on the right knee. In the left knee, the *y*-axis was directed medially (right-handed coordinate system). The harness was positioned on the leg with the sensor directly over the palpated tibial tubercle with its long axis pointing at the second toe and with the foot dorsiflexed. The harness was applied as the last step in the patient setup. Care was taken to position the *y*-axis of the harness/sensor parallel to the table and to the posterior condyles of the distal femur. While the sensor was not directly on the skin, it rested approximately 5 mm from the surface and was consistent side to side.

Both extremities were rotated at the same time into external rotation followed by internal rotation. The motors rotated each leg until the peak torque of 4 Nm was achieved, at which point the direction of rotation was reversed. Three preconditioning cycles were performed, followed by three test cycles with data recording. In addition to the motion measured using the electromagnetic tracking system, rotation of the lower leg as measured at the foot was also collected using an integrated optical encoder at the servomotor. Current was continuously measured and converted into torque (Nm) via an on-board computer program. Motor data was accurate to 0.01° and 0.01 Nm of torque as per the specifications of the motors. No filtering of any kind was performed.

A hysteresis curve was constructed of the three test cycles, with torque on the *y*-axis and rotation on the *x*-axis. Using the loaded portion of the hysteresis curve for each cycle, a third-order polynomial fit of the data was used for analysis. Once fitted, each curve was interpolated for a standard set of 500 torque points between −4 Nm (External Rotation) and +4 Nm (Internal Rotation). No averaging or registration was applied to the data. The position data from the electromagnetic tracking system did not require curve fitting.

The third full load-deformation curve was considered representative of each patient and was used as a single test. Within-test averaging of the multiple cycles was not utilized. Mean load-deformation curves between all patients were constructed using the pointwise mean (i.e. the mean for each of the 500 torque points) of each group along with the pointwise standard error of the mean (SEM). Using the means allowed for pointwise statistical comparison.

### Statistical analysis

Statistical analysis was performed using a custom R program (R: a language and environment for statistical computing, R Foundation for Statistical Computing, Vienna, Austria) to utilize simple functional data analysis (FDA) with pointwise Pearson’s correlation coefficient estimation across the generated curves. Endpoint features including maximum internal rotation, maximum external rotation and foot/tibia position at torque 0 were evaluated using standard sample-based statistical analysis. The position of the tibia and foot at torque 0 Nm represents the position at which the tension in all soft tissues contributing to rotation is zero. This is considered the “equilibrium” position of the limb. Paired data comparisons were utilized when comparing curves constructed from motor position data and curves constructed from the tibial electromagnetic sensor data. A power analysis was performed to test whether a correlation is significantly different from zero using Fisher’s* z* transformation method. A sample size of 33 was shown to provide at least 85 % power at the 0.05 significance level to detect correlations of 0.50 or larger.

## Results

### Demographics

There were 22 males and 10 females included in the data analysis. Patient demographics are shown in Table 1. Three patients had knee recurvatum in the supine position, but this was not measured in degrees. No subject had a healthy knee pivot shift or glide. Three patients had had their injury more than 1 year before surgery.

**Table 1:** Patient demographics for the healthy legs only

	Median value (range)
Age (years)	26 (17–60)
Height (m)	1.8 (1.62–1.90)
Weight (kg)	77.5 (52–100)
Time from injury to surgery (days)	60 (5–3650)
Time from surgery to evaluation (days)	464 (340–672)
Passive knee flexion (prone)	132° (110–144)
Active knee flexion with ankle pull (supine)	148° (130–154)
Passive knee extension (prone)	0° (for all subjects)
KT-1000 manual maximum laxity (absolute) (mm)	7 (4–14)
Thumb laxity (wrist to abducted thumb) (cm)	5 (0–8)
Single-leg triple-hop test (cm)	448.5 (263–648)

### Comparison of foot position and tibial tubercle position

Maximum external rotation of the lower leg as measured at the foot using the encoder count of the motor averaged 49.9° ± 8.1° (range 29.0° to 66.8° ER). Similarly, maximum internal rotation using the same system averaged 14.9° ± 8.0° (range 1.6° ER to 36° IR). One patient was unable to achieve any internal rotation as their natural resting position of the tibia was in extreme external rotation and the toes up position represented their maximum internal rotation. At torque 0 Nm, as measured using the encoder count of the motor, the foot was positioned at an average of 15.1° ± 10.5° of external rotation (range 27.9° ER to 2.1° IR).

At torque 0 Nm, the tubercle was positioned at an average of 11.8° ± 7.1° of internal rotation (range 1.7° ER to 27.2° IR). Maximum external rotation of the tibial tubercle as measured using the electromagnetic tracking system averaged 6.2° ± 7.9° (range 11.9° IR to 20.6° ER). Similarly, maximum internal rotation using the same system averaged 25.6° ± 7.0° (range 10.4° to 43.7° IR).

### Equilibrium position of the foot and tibial tubercle

At torque 0 Nm, the tibial tubercle was positioned an average of 26.9° ± 11.6° (range 5.6° to 48.6° IR) more internally rotated than the foot during lower leg axial rotation.

### Load-deformation curves

**Fig. 2:**
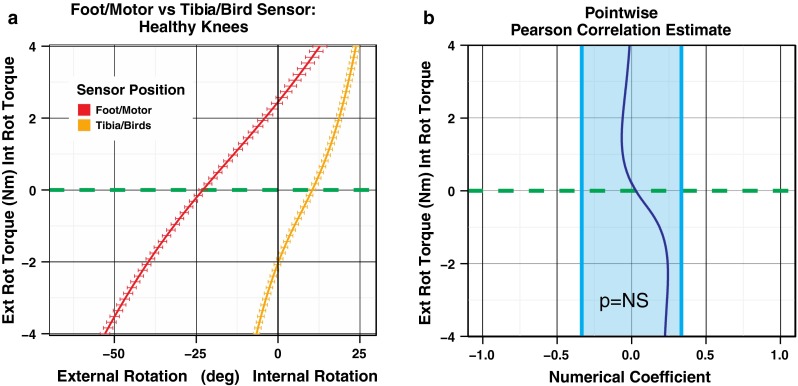
**a** Load-deformation curves comparing lower leg rotation measured at the foot versus tibial rotation measured using a sensor on the proximal tibia. Each load-deformation curve is constructed from the torque of the motor; however, the position/rotation during load is taken either from the motor optical encoder or from the sensor attached to the tibia. **b** Pointwise Pearson’s correlation coefficients indicating no correlation between the load-deformation curves during internal rotation with a mild trend in correlation during external rotation

Figure 2a compares the mean load-deformation curve representing the average position of the foot measured from the encoder count of the motor combined with the torque produced by the motor and the load-deformation curve representing the average position of the tibia as measured using the electromagnetic tracking system combined with the torque produced by the motor. The standard error bars are at every 10 units of torque and represent the variation in position between healthy subjects. Figure 2b shows the Pearson’s correlation coefficient between the load-deformation curves. The Pearson’s correlation coefficient was calculated in a pointwise fashion at each unit of torque (500 units between −4 and 4 Nm). While there was no correlation between the curves during internal rotation, there was a mild trend during external rotation. However, this trend during external rotation does not reach statistical significance.

There are many joints existing between the plantar aspect of the foot and the hip. The most important anatomical joints for axial rotation are the midfoot joints, subtalar joint, talocrural joint and the knee. Motion at all of these joints can contribute to the axial rotation of the foot during a dial test. By dorsiflexing the foot to its maximum position, an attempt is made to reduce the amount of axial rotational “play” at the talocrural joint by placing the widest portion of the talus between the malleoli. In addition, the midfoot and subtalar joints were stabilized by the bottom plate, a supination wedge and straps. All of this was done in order to minimize axial rotation at the foot/ankle in order to insure the best “attempt” to correlate axial rotation at the plantar aspect of the foot and the tibia. A small sample of patients was tested with and without dorsiflexion confirming the significant increase in lower leg axial rotation without forced dorsiflexion (unpublished data). This attempt was performed to show the clinician that even in the best-case situation, the rotation of the foot does not correlate with rotation of the tibia at the knee.

### Off-axis motion

**Fig. 3:**
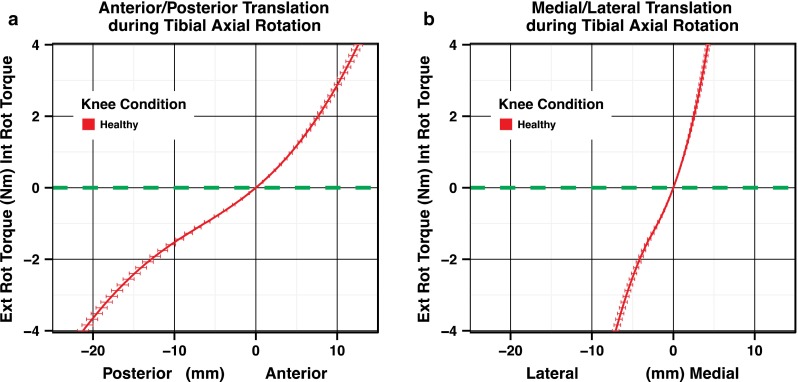
**a** Average load-deformation curve for anterior–posterior translation during tibial axial rotation. **b** The average load-deformation curve for medial–lateral translation during tibial axial rotation (the SEM *error bars* represent the variation in position at each torque unit)

**Fig. 4:**
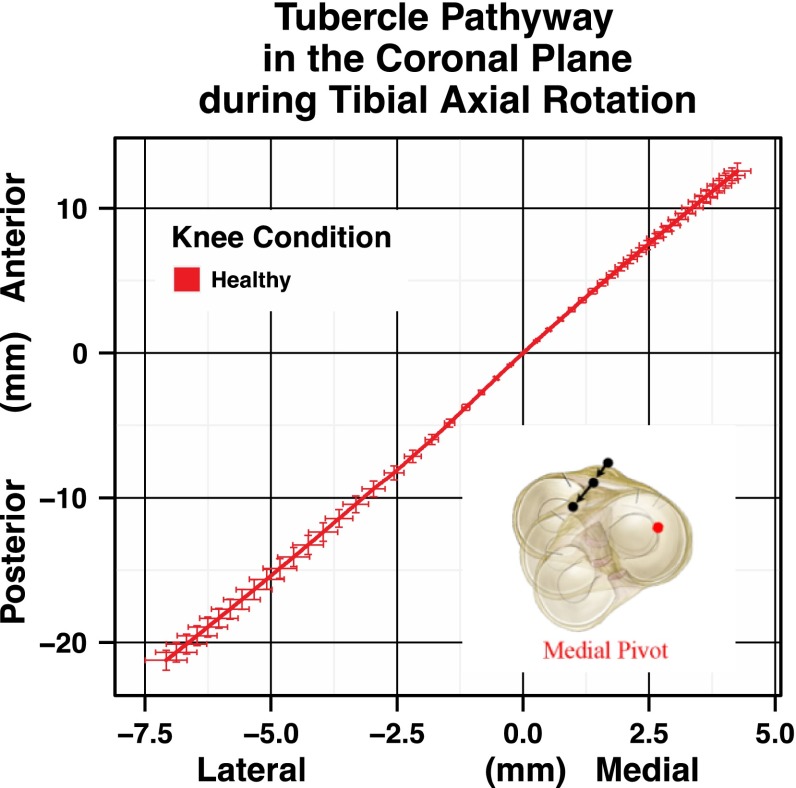
A representation of the relationship between anterior–posterior translation and medial–lateral translation during tibial axial rotation combining the two graphs from Fig. 3 to show the pathway produced by the tibial tubercle in the coronal plane

During tibial axial rotation, the tibial tubercle defines a specific path of motion in the x–y plane or coronal plane. The coronal plane represents lateral to medial translation and posterior to anterior translation. This translation can be represented as a load-deformation curve as in Fig. 3a for posterior to anterior translation or Fig. 3b for lateral to medial translation. The tubercle moves anterior-medial with internal rotation and posterior-lateral with external rotation of the tibia. By combining the posterior to anterior motion and the lateral to medial motion into one *x*–*y* plane graph, Fig. 4 is produced. The curve is constructed from −4 to 4 Nm of torque, and the load dimension is removed. This representation of the data can help to identify changes in the pathway of motion of the proximal tibia during tibial axial rotation.

## Discussion

The most important finding in this study was that a robotic tibial rotation device and an electromagnetic tracking system could accurately mimic the dial test by capturing torque data in addition to the motion of the proximal tibia in all 6° of freedom. Anterior/posterior and medial/lateral translations of the tibia were of primary interest along with axial rotation of the tibia. Compression/distraction, varus/valgus rotation and flexion/extension rotation were measured, but not reported in this study. This system provides significantly more information than the traditional manual dial test that focused on long-axis rotation alone and provides numerical knee laxity data that can mimic the modern dial test. Capturing both the rotation of the tibia as represented by the tibial tubercle and its translation in the coronal (ML-AP) plane is critical in fully characterizing the motion of the proximal tibia. This information allows for increased accuracy in making a diagnosis of a knee injury.

The use of the dial test as a means of diagnosing rotational injuries to the knee has been discussed in the literature since it was developed from the posterolateral drawer test as described by Hughston and Norwood [19]. There are many ways in which this test has been reproduced with the main focus on increased external rotation on the injured side as the key measure of potential PLC injury. Pearle and others have abandoned the use of rotation alone and have incorporated lateral compartment translation as the key measure [12, 25]. The use of the electromagnetic tracking system during the dial test allows for a measure of proximal tibial translation. The combination of rotational and translational load-deformation curves describing the motion of the proximal tibia should add to the surgeon’s clinical database in determining the presence of rotational instability.

The second important finding in this study was that the use of the foot as a measure of tibial axial rotation, and thus, in the production of a tibial rotational load-deformation curve will lead to inaccuracies. The traditional dial test uses the foot–thigh angle to determine tibial axial rotation at the knee. Unfortunately, the position of the foot in internal/external rotation is not correlated with the position of the tibial tubercle. This finding matches the results of previous studies that have shown that the rotation of the foot is not an accurate measure tibial rotation [2, 3, 26]. This does not mean that lower leg rotation as measured by foot position is not valuable. Foot position and its concomitant load-deformation curve have value in studying the leg as a system [9, 11]. However, foot position cannot be used to indicate tibial position with respect to the femur.

**Fig. 5:**
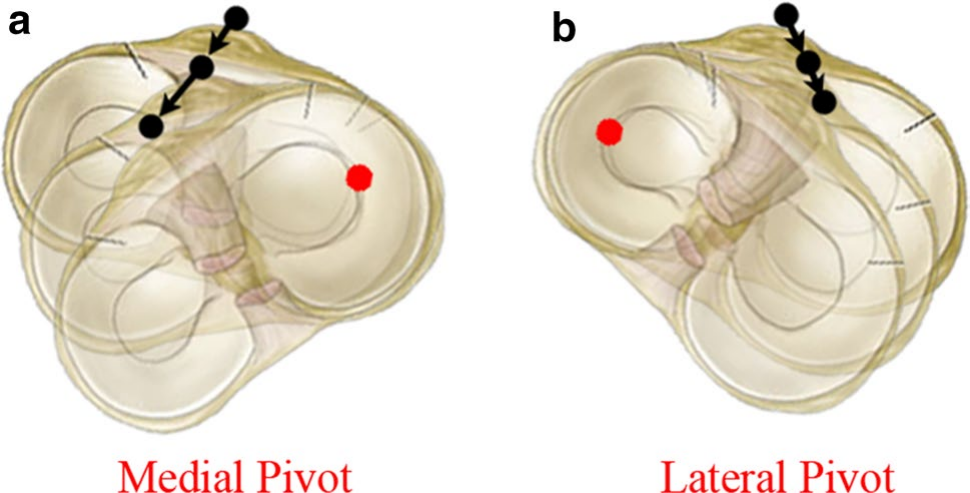
**a** A representation of the tubercle pathway of a “medial pivot”. **b** A representation of the pathway of a “lateral pivot”

The third important finding in this study is that the motion of the tibial tubercle in the coronal (ML-AP) plane helps define the location of a “centre of rotation” or a general “pivot point” within or near the proximal tibia during tibial axial rotation. The shape of the tibial tubercle pathway and its direction give insight into the condition of the ligaments influencing this pathway during rotation. For example, if the main ligament of rotational influence is the medial collateral ligament, then the proximal tibia is likely to rotate about a “medial pivot” (Fig. 5a). On the other hand, if the medial collateral ligament is damaged, perhaps the proximal tibia will rotate about a “lateral pivot” (Fig. 5b). The pathway data from the motion of the tubercle suggest that the proximal tibia does not rotate about a single axis.

When examining the knee for ligament injuries, the extent of motion in the injured knee is often compared to the extent of the opposite or healthy knee to determine if an injury has occurred. This dependence upon side-to-side comparison can be problematic for without a “healthy” knee the clinician has no frame of reference for analysis of the injured knee. Diagnosis in bilateral patients is extremely difficult, and in some patients who report one healthy knee, that knee may not truly be healthy but rather only asymptomatic. The knee itself is a system and has the capability of being stable or unstable like any other mechanical system. This stability can be measured in each limb individually. Currently, the pivot shift test is the best clinical indicator of a knee that is unstable or at least has the potential of being unstable during activity. It does not require comparison between knees for the test to be positive. Despite attempts to standardize the pivot shift test through either training or instrumentation, consistency and reproducibility of the in vivo test have remained low [18, 20, 23]. An understanding of the three-dimensional motion of the proximal tibia during application of tibial axial torque may be able to provide the consistency and reproducibility necessary to produce a single-limb test indicating potential knee instability. This could possibly be achieved through analysis of the shape of the pattern of motion seen in the coronal plane (ML-AP), which can be performed in a unilateral knee. This technique has not been validated, but may be explored in future studies.

This study included analysis of the healthy knees of a small sample of subjects from a population of patients requiring an ACL reconstruction. As a result, it may not be representative of the population as a whole. It has been reported that tibiofemoral kinematics can be altered in the opposite, healthy knee of ACL-reconstructed patients post-surgery [17]. In this study, the reported kinematics may not be representative of those seen in patients with bilateral healthy knees. The results in this study were limited by the setup of the patient’s foot in the device, the ability of the device to capture the distal femur and limit its rotation, and the measurement accuracy of the system. The electromagnetic tracking system was placed on the tibial tubercle and tibial crest in a consistent fashion side-to-side and from patient to patient, but its position was not calibrated to a world coordinate system nor was it calibrated to each individual anatomical malleolar axis. The x–y or coronal plane (ML-AP) translation was registered to the torque 0 position identified during tibial axial rotation. It was assumed that this position had subject-to-subject consistency in the healthy limb. The position of the tibial tubercle as measured in this study was approximately 5 mm anterior to its anatomical location.

The dial test performed by the clinician should focus observation at the tibial tubercle and not on the foot. During the clinical dial test, a mark should be made on the patient’s tibial tubercle with observations of that mark during axial rotational force application at the foot. The motion of the tubercle itself should give information as to increased rotation/translation of the proximal tibia. Certainly, during the test, motion of the medial and lateral tibial plateaus can be observed with additional information in regard to their positional change. The main point to be made is that clinical observation of the foot during the test tells the clinician something completely different than clinical observation of the proximal tibia. Pearle et al. and Branch et al. [7, 25] have shown that posterior translation of the lateral tibial plateau during the dial test is more sensitive for PLC instability than rotation alone.

## Conclusions

The clinical information captured by a surgeon during a dial test includes both rotation of the tibia and proximal tibia translation during load application. All of this information can be captured using a robotic tibial axial rotation device with an electromagnetic tracking system and a sensor on the proximal tibia. The translation of the proximal tibia is important information that must be considered in addition to axial rotation of the tibia when performing a dial test whether done manually or with a robotic device. Instrumented foot position cannot provide the same information. While foot/ankle rotation is not a true representation of proximal tibial rotation during the dial test, it can provide valuable information for the analysis of the lower leg as a system. Finally, the pathway of the tibial tubercle during tibial axial rotation can provide additional information about knee instability without relying on side-to-side comparison between knees.
